# Multiscale computational analysis of the steady fluid flow through a lymph node

**DOI:** 10.1007/s10237-024-01879-7

**Published:** 2024-09-25

**Authors:** Alberto Girelli, Giulia Giantesio, Alessandro Musesti, Raimondo Penta

**Affiliations:** 1 Dipartimento di Matematica e Fisica “N. Tartaglia”, Università Cattolica del Sacro Cuore, Brescia, Italy; 2 Mathematics for Technology, Medicine and Biosciences, Università degli Studi di Ferrara, Ferrara, Italy; 3 School of Mathematics and Statistics, University of Glasgow, Glasgow, UK

**Keywords:** Multiscale modelling, Lymph flow, Numerical simulations, Physiological data

## Abstract

Lymph Nodes (LNs) are crucial to the immune and lymphatic systems, filtering harmful substances and regulating lymph transport. LNs consist of a lymphoid compartment (LC) that forms a porous bulk region, and a subcapsular sinus (SCS), which is a free-fluid region. Mathematical and mechanical challenges arise in understanding lymph flow dynamics. The highly vascularized lymph node connects the lymphatic and blood systems, emphasizing its essential role in maintaining the fluid balance in the body. In this work, we describe a mathematical model in a steady setting to describe the lymph transport in a lymph node. We couple the fluid flow in the SCS governed by an incompressible Stokes equation with the fluid flow in LC, described by a model obtained by means of asymptotic homogenisation technique, taking into account the multiscale nature of the node and the fluid exchange with the blood vessels inside it. We solve this model using numerical simulations and we analyze the lymph transport inside the node to elucidate its regulatory mechanisms and significance. Our results highlight the crucial role of the microstructure of the lymph node in regularising its fluid balance. These results can pave the way to a better understanding of the mechanisms underlying the lymph node’s multiscale functionalities which can be significantly affected by specific physiological and pathological conditions, such as those characterising malignant tissues.

## Introduction

Lymph Nodes (LNs) are essential components of the lymphatic system, acting as filters that eliminate harmful substances like bacteria, viruses, and waste products. The lymph node plays a crucial role in both the immune and lymphatic systems, serving as a vital component in safeguarding the body against infections and diseases and regulating lymph transport. The main immunological function of the lymph node is achieved by hosting lymphocytes, such as B and T cells, which travel through the bloodstream and reside in the nodes. B cells generate antibodies that specifically attach to antigens, triggering an immune response. When activated, B cells can differentiate into plasma cells that release antibodies or memory cells that provide defense in subsequent encounters. Additionally, specialized antigen-presenting cells (APCs), like dendritic cells (DCs), capture and process antigens from diverse sources. These cells then migrate to the lymph nodes, presenting the antigens to T cells, activating them, and kickstarting the adaptive immune response.

Interstitial fluid, known as *lymph* once it enters the lymphatic system, plays a crucial role in transporting immune cells, proteins, cancer cells, drugs, and other substances (O’Melia et al 2019; Arasa et al 2021; Birmingham et al 2020; Apoorva et al 2018; Permana et al 2021). When lymph transport is compromised, it can result in lymphœdema, a condition characterized by an abnormal accumulation of fluid in the tissues. Lymph nodes play a major role in regulating lymph transport: indeed one of the causes of lymphœdema is the excision and removal of lymph nodes (Moore Jr and Bertram 2018; Tobbia et al 2009). The lymph transport within the node is important from a biological point of view, but it also presents some interesting mathematical and mechanical challenges. From a mechanical perspective, the lymph node comprises two primary components: the lymphoid compartment (LC), which forms the porous bulk region of the node, and the subcapsular sinus (SCS), a narrow free-fluid channel located near the wall that surrounds the LC (Margaris and Black 2012). The lymph can permeate the LC from the SCS through a network of conduits established by fibroblastic reticular cells (FRC) that form the porous structure of the node (Novkovic et al 2020; Grebennikov et al 2016; Savinkov et al 2017). Initially, the lymph flows inside the subcapsular sinus of the node, and then a part of the lymph goes into the lymphoid compartment and the remaining part (the majority) leaves the node. The dendritic cells and macrophages present in the lymph are transported at the interaction surface between the SCS and the LC, initiating the immune response. The lymph node is a highly vascularized organ, and inside the LC compartment, there are blood vessels that allow the exchange of fluid and substances, making the LN an important connection between the lymphatic and the blood system.

The movement of lymph within the lymph node is a highly significant and intriguing physical process. It involves a complex multiscale architecture with an intricate microenvironment and the interplay between the free-fluid region in the subcapsular sinus and the porous lymphoid compartment. Moreover, this process integrates interactions between the lymphatic and blood systems, crucial for immune surveillance and response. Understanding these dynamics is pivotal, as deviations can lead to various pathologies, from lymphœdema to cancer metastasis. Thus, the lymph node serves as a connection where fluid dynamics, immune function, and physiological intricacies converge, shaping our understanding of health and disease. However, yet, only a few mathematical models in literature explore it (Novkovic et al 2018; Shanti et al 2018; Jayathungage Don et al 2023). An image-based modeling approach to obtain data regarding the internal structure of the lymph node is proposed in Cooper et al (2016, 2018), where they used these data to find the permeability of a Darcy equation used to describe the lymph flow in the whole node. Another computational flow model is studied in Jafarnejad et al (2015), in which they study a mouse popliteal LN in an idealized spheroidal geometry, differentiating the fluid flow in the SCS (using a Navier-Stokes equation) and the fluid flow in the LC (using a Darcy-Brinkman equation). Setukha and Tretiakova (2022) propose numerical simulation using boundary integral equations to simulate the fluid flow in the lymph node. In the literature, some more computational models describe the fluid flow in a lymph node. In Tretiakova et al (2021) they develop an artificial neural network model based on Setukha and Tretiakova (2022) and on the experimental results of Adair et al (1982); Adair and Guyton (1983, 1985) to describe the lymph node drainage function. A three-dimensional geometry of the fibroblastic reticular cell graph network generated by an object-oriented computational algorithm is developed in Grebennikov et al (2016); Savinkov et al (2017) to study the lymph flow through the conduit system network. Another interesting approach used to describe the fluid flow within the node is to use a microfluidic platform, like in Shanti et al (2020) the authors simulate the fluid flow in a microenvironment mimicking the lymph node properties; another microfluidic platform that recreates the lymph node’s subcapsular sinus microenvironment is developed by Birmingham et al (2020), where they investigate how physiological flow patterns impact the adhesion of metastatic cancer cells. All the papers presented above have a computational and experimental nature; in Giantesio et al (2021, 2022) we have the first attempts to analytically study the lymph movement within the lymph node. An analytical and a numerical solution are presented in a time-dependent setting in simplified geometries (a cylindrical geometry in Giantesio et al (2021) and a spherical geometry in Giantesio et al (2022)), without considering the drainage of the blood vessels. In particular, in Giantesio et al (2022) we coupled the lymph flow in the subcapsular sinus with the flow in the lymphoid compartment using stream functions, without considering the blood vessels and the fluid exchange within them. The drainage function of the blood vessels inside the node and the multiscale nature of the latter are considered in Girelli et al (2023), obtaining a rigorous mathematical model using the asymptotic homogenization technique (Gerisch et al 2018; Hornung 1997; Auriault et al 2009) describing the fluid flow inside both the FRC and the blood vessels networks, without considering the subcapsular sinus, in a steady setting. We found an analytical solution, in a simplified spherical geometry, which describes the fluid flow and the fluid exchange between the FRC and the blood vessels. In this work, we extend the results of our previous work Girelli et al (2023) taking into account the fluid flow in the subcapsular sinus, coupling this flow with the flow inside the lymphoid compartment in a more realistic geometry, giving detailed results for the entire lymph node.

In Sect. 2, we recall the steady mathematical model that describes the fluid flow inside both the subcapsular sinus and the lymphoid compartment. In Sect. 3 we describe the numerical simulations used to solve the steady problem and we describe the results using physiological parameters obtained from the lymph node literature. In particular, in Sect. 3.1 we study the steady lymph flow in a spherical geometry and we compare the results with the analytical founding of Girelli et al (2023), and Sect. 3.2 is devoted to the steady lymph flow in an oblate spheroidal geometry, which is a more realistic geometry for a lymph node (Jafarnejad et al 2015; Tretiakova et al 2021; Margaris and Black 2012; Giantesio et al 2021; Shanti et al 2020; O’Melia et al 2019).

## Mathematical model

**Fig. 1:**
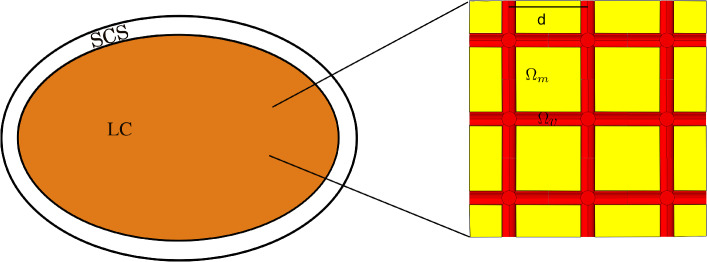
A 2D representation of the microscale and macroscale geometries. On the left-hand side, there is a sketch of the macroscale of the lymph node. On the right-hand side, there is the periodic cell representing the microscale

In this section, we introduce the mathematical model that we use to describe the fluid flow inside the lymph node in a steady setting. In Fig. 1 we can see a sketch of the geometry of our problem, showing the subcapsular sinus (SCS), the lymphoid compartment (LC), and, on the right-hand side, the microstructure of the conduit system network, formed by FRC lymph conduits represented by the geometry $\Omega _m$ and the blood vessels represented by $\Omega _v$. We emphasize that the cylinders forming the geometry $\Omega _v$ physically represent vascularized regions rather than individual vessels, as described in Girelli et al (2023).

We suppose that the lymph is an incompressible Newtonian fluid similar to water (Moore Jr and Bertram 2018) so that the fluid in the subcapsular sinus can be described by the steady Stokes equation due to the small velocity and small characteristic length

1 $$ \left\{ {\begin{array}{*{20}l}    {\mu \Delta \varvec{u}^{f}  = \nabla p^{f} ,} \hfill  \\    {\nabla  \cdot \varvec{u}^{f}  = 0,} \hfill  \\   \end{array} } \right. $$

where $\mu$ is the viscosity, $\varvec{u}^f$ is the velocity in the SCS, and $p^f$ is the pressure in the SCS.

For the lymphoid compartment (porous bulk region of the lymph node) we use the model proposed in Girelli et al (2023) to describe the fluid flow and the fluid exchange between the lymph and the blood vessels. Here we summarize the model; see Girelli et al (2023) for more details about its derivation.

First of all, we define the average operator as

2 $$\begin{aligned} \langle h\rangle _{\Omega _\gamma }=\dfrac{1}{|\Omega _\gamma |} \int _{\Omega _\gamma }h d\varvec{y}, \quad \gamma =m, v, \end{aligned}$$

where $\varvec{y}$ is the variable that describes the microscale problem, which is connected to the macroscale variable $\varvec{x}$ by the relationship $\varvec{y}=\varvec{x}/\epsilon$, with

$$\begin{aligned} \epsilon =d/L\ll 1, \end{aligned}$$

*d* is the *microscale characteristic length* related to the distance of the centers of the cylinders in the cell domain of Fig. 1 (*d* physically represents the distance between two vascularized regions) and *L* is the *macroscale characteristic length*.

The macroscopic model of the fluid flow for the FRC phase $\Omega _m$ is as follows

3 $$\begin{aligned} \langle \varvec{u}_m^{(0)}(\varvec{x},\varvec{y})\rangle _{\Omega _m}=-\frac{d^2}{\mu }\langle \varvec{W}_m(\varvec{x},\varvec{y})\rangle _{\Omega _m} \nabla _{\varvec{x}} p_m^{(0)}(\varvec{x}), \end{aligned}$$

4 $$\begin{aligned} \nabla _{\varvec{x}} \cdot \langle \varvec{u}_m^{(0)} (\varvec{x},\varvec{y})\rangle _{\Omega _m} = \nonumber \\ -\dfrac{L_p S^\text {tot}}{|\Omega _m^\text {tot}|}\left[ p_m^{(0)}(\varvec{x})-p_v^{(0)}(\varvec{x})-\sigma \left( \pi _m-\pi _v\right) \right] , \end{aligned}$$

where $\varvec{u}_m^{(0)}$ and $p_m^{(0)}$ are the leading-order velocity and pressure of the asymptotic homogenization expansion presented in the model of Girelli et al (2023) of the phase $\Omega _m$, respectively; $L_p$ is a quantity that describes the geometry and the tissue wall material at the intersection between the two phases described in ms^−1^ Pa^−1^ (Waniewski 2006), $S^{\rm tot}$ is the total blood vessels surface, $|\Omega _m^{\rm tot}|$ is the total volume of the phase $\Omega _m$, $\sigma$ is the *Staverman’s reflection coefficient* that describes the leakiness of the capillary membrane to proteins, $\pi _v$ and $\pi _m$ are the *oncotic pressure of phase* $\Omega _v$ and $\Omega _m$, respectively, and $\varvec{W}_m$ is a second-order tensor obtained as the solution of the following cell problem (obtained by the asymptotic homogenization technique in Girelli et al 2023):

5 $$  \left\{ {\begin{array}{*{20}l}    {\varvec{K}_{m}^{{ - 1}} \left( {\varvec{x},\varvec{y}} \right)\varvec{W}_{m} \left( {\varvec{x},\varvec{y}} \right) - \mu ^{*} \Delta _{\varvec{y}} \varvec{W}_{m} \left( {\varvec{x},\varvec{y}} \right) - \mathbb{I} + \left( {\nabla _{\varvec{y}} \varvec{g}_{m} \left( {\varvec{x},\varvec{y}} \right)} \right)^{T}  = {\mathbf{0}}} \hfill & {{\text{in}}\;\Omega _{m} ,} \hfill  \\    {\nabla _{\varvec{y}}  \cdot \varvec{W}_{m} \left( {\varvec{x},\varvec{y}} \right) = {\mathbf{0}}} \hfill & {{\text{in}}\;\Omega _{m} ,} \hfill  \\    {\varvec{W}_{m}^{T} \left( {\varvec{x},\varvec{y}} \right)\varvec{n} = {\mathbf{0}}} \hfill & {{\text{on}}\;\Gamma ,} \hfill  \\    {\varvec{W}_{m}^{T} (\varvec{x},\varvec{y})\varvec{\tau } =  - \frac{{\sqrt {\varvec{K}_{m} \left( {\varvec{x},\varvec{y}} \right)} }}{\alpha }\left[ {\left( {\nabla _{\varvec{y}} \varvec{W}_{m}^{T} \left( {\varvec{x},\varvec{y}} \right)} \right)\varvec{n}} \right]\varvec{\tau }} \hfill & {{\text{on}}\;\Gamma ,} \hfill  \\    {\left\langle {\varvec{g}_{m} \left( {\varvec{x},\varvec{y}} \right)} \right\rangle _{{\Omega _{m} }}  = {\mathbf{0}}} \hfill & {{\text{in}}\;\Omega _{m} .} \hfill  \\   \end{array} } \right.   $$

Here $\Gamma$ is the interface between the phases $\Omega _m$ and $\Omega _v$, $\varvec{n}$ is the outer normal to $\Omega _m$, $\tau$ any tangential vector to the interface $\Gamma$, ${\mathbb {I}}$ is the second-order identity tensor, $\varvec{K}_m$ is the hydraulic conductivity of the phase $\Omega _m$, $\mu ^*$ is the ratio between the effective viscosity $\mu _e$ (Brinkman 1949) and the fluid viscosity $\mu$, $\alpha$ is the Beavers-Joseph-Saffman parameter (Beavers and Joseph 1967; Saffman 1971), and $\varvec{g}_m$ is a vector obtained exploiting the asymptotic homogenization technique in Girelli et al (2023). The last equation of system (5) ensures the uniqueness of the solution.

For the macroscopic model of the fluid flow for the blood vessel phase $\Omega _v$, we have

6 $$\begin{aligned}{} & {} \begin{aligned}&\langle \varvec{u}_v^{(0)}(\varvec{x},\varvec{y})\rangle _{\Omega _v}=\\ {}&-\frac{d^2}{\mu }\langle \varvec{K}_v(\varvec{x},\varvec{y})\left( {\mathbb {I}} +(\nabla _{\varvec{y}}\varvec{g}_v(\varvec{x},\varvec{y}))^T\right) \rangle _{\Omega _v}\nabla _{\varvec{x}}p^{(0)}_v(\varvec{x}) \end{aligned}\end{aligned}$$

7 $$\begin{aligned}{} & {} \begin{aligned}&\nabla _{\varvec{x}} \cdot \langle \varvec{u}_v^{(0)} (\varvec{x},\varvec{y})\rangle _{\Omega _v} =\\ {}&\dfrac{L_p S^\text {tot}}{|\Omega _v^\text {tot}|}\left[ p_m^{(0)}(\varvec{x})-p_v^{(0)}(\varvec{x})-\sigma \left( \pi _m-\pi _v\right) \right] , \end{aligned} \end{aligned}$$

where $\varvec{K}_v$ is the *hydraulic conductivity* of the phase $\Omega _v$, $\varvec{u}_v^{(0)}$ and $p_v^{(0)}$ are the leading-order velocity and pressure of the asymptotic homogenization expansion presented in the model of Girelli et al (2023) of the phase $\Omega _v$, respectively; $\varvec{g}_v$ is a vector obtained by the solution of the following cell problem (obtained by the asymptotic homogenization technique in Girelli et al (2023)):

8 $$\begin{aligned} {\left\{ \begin{array}{ll} \nabla _{\varvec{y}}\cdot \left[ \nabla _{\varvec{y}}\varvec{g}_v(\varvec{x},\varvec{y})\varvec{K}_v(\varvec{x},\varvec{y})^T\right] =\\ -\nabla _{\varvec{y}}\cdot \varvec{K}_v(\varvec{x},\varvec{y})^T, \quad &{} \text {in} \ \Omega _v \\ \left[ \nabla _{\varvec{y}}\varvec{g}_v(\varvec{x},\varvec{y})\varvec{K}_v(\varvec{x},\varvec{y})^T\right] \cdot \varvec{n}=\\ -\varvec{K}_v(\varvec{x},\varvec{y})^T\cdot \varvec{n}\quad &{} \text {on} \ \Gamma ,\\ \langle \varvec{g}_v(\varvec{x},\varvec{y})\rangle _{\Omega _v}=\varvec{0} \quad &{} \text {in} \ \Omega _v. \end{array}\right. } \end{aligned}$$

Again, the last equation of system (8) ensures the uniqueness of the solution.

We assume to have an afferent lymphatic vessel at the upper part of the lymph node and an efferent lymphatic vessel at the lower part. The boundary conditions that we impose are: uniform flow velocity $v_\text {in}$ as inlet condition in the upper lymphatic vessel, the pressure $p_\text {out}$ as outlet condition in the lower lymphatic vessel, no-slip condition at the external wall. At the macroscopic interface $\Gamma _M$ between the free fluid region (SCS) and the porous region (LC) we impose the following interface conditions (Discacciati and Quarteroni 2009)

9 $$\begin{aligned} \varvec{u}^f\cdot \varvec{n}^M=\langle \varvec{u}_m^{(0)}\rangle _{\Omega _m}\cdot \varvec{n}^M\end{aligned}$$

10 $$\begin{aligned} -\left( \varvec{T}(\varvec{u}^f,p^f) \varvec{n}^M\right) \cdot \varvec{n}^M=p_m^{(0)}\end{aligned}$$

11 $$\begin{aligned} \varvec{u}^f\cdot \varvec{\tau }^M_j = -\dfrac{\sqrt{\varvec{{\mathcal {K}}}_m}}{\alpha _M}\left[ \left( \varvec{n}^M \cdot \nabla \right) \varvec{u}^f\right] \cdot \varvec{\tau }^M_j \end{aligned}$$

where $\varvec{T}$ is the Cauchy stress tensor of the free-fluid region, $\varvec{{\mathcal {K}}}_m$ is the macroscopic permeability (obtained from the cell problem (5) of the phase $\Omega _m$, that in our specific case is constant due to the isotropy of the porous medium, i.e. $\varvec{{\mathcal {K}}}_m(\varvec{x})\equiv {\mathcal {K}}{\mathbb {I}}$, where ${\mathbb {I}}$ is the second order identical tensor), $\alpha _M$ is a parameter that needs to be estimated and depends on the physicochemical properties of the interface (Irons et al 2017), $\varvec{n}^M$ is the normal vector related to $\Gamma _M$, and $\varvec{\tau }^M_j$ for $j=1,2$ are the tangents related to the normal $\varvec{n}^M$. The last interface condition is the so-called *Beavers-Joseph-Saffman boundary condition* (BJS). The BJS is an interface condition formulated experimentally in Beavers and Joseph (1967); Saffman (1971).

Equation (11) can be simplified letting $\alpha _M \rightarrow \infty$, which gives (Discacciati and Quarteroni 2009; Auriault 2010)

12 $$\begin{aligned} \varvec{u}^f \cdot \varvec{\tau }_j = 0 \quad \text {on} \ \Gamma _M, \quad j=1,2. \end{aligned}$$

Using (12) in place of (11) we get a difference of about $\epsilon$ with respect to the whole BJS (Discacciati and Quarteroni 2009).

### Remark 1

We note that the Beavers-Joseph-Saffman boundary conditions were found experimentally in Beavers and Joseph (1967); Saffman (1971) and demonstrated in Jäger and Mikelić (2000, 2009), but only in a 2D laminar case (as mentioned in Auriault (2010)), and the extension to a generic geometry is non-trivial (Eggenweiler and Rybak 2021; Shipley and Chapman 2010). Moreover, in Auriault (2010) they employ an asymptotic homogenization expansion to study the interface between a free-fluid region and a porous region, and they found that the simplified boundary condition (12) is also valid for correctors of order higher than $\epsilon$. For these reasons, for most of the paper, we will consider the simplified boundary condition (12), although some comparisons with the BJS boundary condition are made for the sake of completeness.

## Numerical simulations

In this section we solve numerically the macroscopic flow related to the model described in the previous section, aimed at coupling the motion of the flow in the subcapsular sinus (SCS) and the lymphoid compartment (LC) in a steady setting. Indeed, in Girelli et al (2023), we supposed a given pressure distribution for the SCS and we imposed this pressure as a boundary condition for the porous bulk region (the LC). However, in general, we need to couple these two domains.

The physiological data are the same as in Girelli et al (2023, Appendix B), and they are summarized in Table 1. The cell problems (5) and (8) are solved using COMSOL Multiphysics in the same way as we did in Girelli et al (2023, Appendix C); the solution method is given in Sect. 4 for the reader’s convenience.

**Table 1:** Physiological and estimated parameters. For a complete review, we refer to Girelli et al (2023, Appendix B)

Name	Physiological Range/Value	Description
*R*	0.49 mm	Macroscopic radius (Birmingham et al 2020; Giantesio et al 2022)
*a*, *b*	0.5 mm, 0.35 mm	Major and minor spheroidal semiaxes (Jafarnejad et al 2015)
*h*	0.01 mm	Subcapsular sinus height (Jafarnejad et al 2015; Ohtani and Ohtani 2008)
$\mu$	$1 \,\frac{\text{ mg }}{\text{ mm } \text{ s }}$	Viscosity (Moore Jr and Bertram 2018; Bertram et al 2017)
$\phi$	0.75	Porosity (Shanti et al 2020)
$\mu _e$	$\frac{\mu }{\phi }$	Effective viscosity (Ochoa-Tapia and Whitaker 1995a, b; Brinkman 1949; Tan and Pillai 2009)
$\rho _0$	$1 \,\frac{\text{ mg }}{\text{ mm}^3}$	Density (Moore Jr and Bertram 2018; Bertram et al 2017)
${\hat{K}}_m$	$3.84 \times 10^{-9}$ mm^2^	Permeability of the interstitium (Shanti et al 2020; Savinkov et al 2017)
$\sigma$	$0.88-0.9$	Staverman’s coefficient (Jafarnejad et al 2015; Cooper et al 2016, 2018; Tretiakova et al 2021)
$\pi _v-\pi _m$	$3.41\times 10^5-2.08\times 10^6$ mPa	Oncotic pressure difference (Jafarnejad et al 2015; Cooper et al 2016, 2018; Tretiakova et al 2021; Adair et al 1982; Adair and Guyton 1983, 1985; Stohrer et al 2000)
$L_p$	$5.475\times 10^{-12}-3.67\times 10^{-8}\,\frac{\text{ mm }}{\text{ s } \text{ mPa }}$	Hydraulic conductivity of the blood vessel walls(Jafarnejad et al 2015; Cooper et al 2016, 2018; Tretiakova et al 2021)
${\bar{p}}_v$	$6.67\times 10^5-1.066\times 10^6$ mPa	Mean blood vessel pressure (Jafarnejad et al 2015; Cooper et al 2016, 2018; Tretiakova et al 2021)
$S^\text {tot},|\Omega _v^\text {tot}|$	13.4 mm^2^, 0.0322 mm^3^	Blood vessel surface and volume (Jafarnejad et al 2019; Kelch et al 2015)
*N*	1310	Number of cells (Girelli et al 2023, Appendix B)
$r_c,d$	$1.7\times 10^{-3}$ mm, $2\times 10^{-2}$ mm	Microscale cylinders radius and mean distance (Girelli et al 2023)
*L*	1 mm	Coarse scale characteristic length
$K_v\dfrac{d^2}{\mu }$	$1.1 \times 10^{-6}\,\frac{\text{ mm}^3\text{ s }}{\text{ mg }}$	Hydraulic conductivity of the blood vessels computed using the Kozeny-Carman formula (Kozeny 1927; Carman 1997; Girelli et al 2023)
${\bar{K}}_m$	$3.65\times 10^{-9}\,\frac{\text{ mm}^3 \text{ s }}{\text{ mg }}$	Macroscopic interstitial hydraulic conductivity (solving system (5))
${\bar{K}}_v$	$4.12 \times 10^{-7}\,\frac{\text{ mm}^3 \text{ s }}{\text{ mg }}$	Macroscopic blood hydraulic conductivity (solving system (8))
$v_\text {in}$	$0.22{\mkern 1mu} \frac{{{\text{mm}}}}{{\text{s}}}$	Inlet velocity (Blatter et al 2016)
$\alpha$	1	Beavers-Joseph-Saffman parameter of the cell problem (5)

Here we discuss the weak formulation in the general case of the boundary conditions (9)– (11). Consider a test function

$$\begin{aligned} \varvec{w}\in W_g=\{ \varvec{w}\in H^1 (\Omega ): \varvec{w}_{\Gamma _D}=\varvec{g}\}, \end{aligned}$$

where $\Omega$ is the domain of the problem, $H^1(\Omega )$ is the usual Sobolev space, and $\Gamma _D$ is the portion of the boundary where we have the Dirichlet boundary condition $\varvec{u}^f_{|\partial \Gamma _D}=\varvec{g}$; by using the classical weak formulation of the Stokes equation (1), we can focus on the boundary term of this weak form $-\int _{\Gamma _M}\varvec{n}\cdot \varvec{T}(\varvec{u}^f,p^f) \ \varvec{w}$, so that the weak formulation of the interface conditions (9-11) can be written as (Discacciati and Quarteroni 2009)

13 $$\begin{aligned}&-\int _{\Gamma _M}\varvec{n}\cdot \varvec{T}(\varvec{u}^f,p^f) \ \varvec{w}=\nonumber \\ {}&-\int _{\Gamma _M}\left[ \varvec{n}\cdot {\varvec{T}}(\varvec{u}^f,p^f) \cdot \varvec{n}\right] \varvec{w}\cdot \varvec{n}\nonumber \\ {}&- \int _{\Gamma _M}\sum _{j=1}^{2}\left[ \varvec{n}\cdot \varvec{T}(\varvec{u}^f,p^f)\cdot \varvec{\tau }_j\right] \varvec{w}\cdot \varvec{\tau }_j, \end{aligned}$$

and hence we have, using (10) and (11)

14 $$\begin{aligned}&-\int _{\Gamma _M}\varvec{n}\cdot \varvec{T}(\varvec{v}^f,p^f) \ \varvec{w}=\int _{\Gamma _M}p_m^{(0)}(\varvec{w}\cdot \varvec{n})\nonumber \\+&\int _{\Gamma _M}\sum _{j=1}^{2}\dfrac{\mu \alpha _M}{\sqrt{{\mathcal {K}}}}\left( \varvec{u}^f \cdot \varvec{\tau }_j\right) \left( \varvec{w}\cdot \varvec{\tau }_j\right) =\nonumber \\ {}&\int _{\Gamma _M}(p_m^{(0)} \cdot \varvec{n})\varvec{w}\nonumber \\+&\int _{\Gamma _M}\sum _{j=1}^{2}\left[ \dfrac{\mu \alpha _M}{\sqrt{{\mathcal {K}}}}\left( \varvec{u}^f \cdot \varvec{\tau }_j\right) \cdot \varvec{\tau }_j\right] \varvec{w}. \end{aligned}$$

We use the finite element method to solve numerically the Stokes equation and the macroscopic model given in Sect. 2 using COMSOL Multiphysics. To have more information about the weak formulation of the Stokes equation, we refer to Formaggia et al (2009); Giantesio et al (2022). We implement this flow in COMSOL using the creeping flow module for the Stokes equation (1), with the Taylor-Hood element ${\mathbb {P}}_2^3-{\mathbb {P}}_1$; this means that, given a triangulation ${\mathcal {T}}$ of the domain $\Omega$, we approximate the velocity and the pressure with the piecewise polynomial spaces ${\mathbb {P}}_2^3=\left( {\mathcal {P}}_2({\mathcal {T}})\right) ^3 \cap H_0^1(\Omega )$ and ${\mathbb {P}}_1={\mathcal {P}}_1({\mathcal {T}})\cap L_0^2(\Omega )$, respectively, where ${\mathcal {P}}_k({\mathcal {T}})=\{g \in {\mathcal {C}}(\Omega ):g_{{\textsf{T}}}\in {\mathbb {P}}_2, \forall {\textsf{T}} \in {\mathcal {T}}\}$, $H_0^1(\Omega )=\{w \in H^1(\Omega ): w_{|\partial \Omega }=0\}$, $L_0^2(\Omega )=\{w \in L^2(\Omega ): w_{|\partial \Omega }=0\}$, $H^1(\Omega )$ is a Sobolev space and $L^2(\Omega )$ is a Banach space. To implement the boundary condition (14), we use the general stress boundary condition of COMSOL. Moreover, from equations (3), (4), (6), and (7), we have that the Darcy problems can be written as diffusion problems for the pressure and we refer to Quarteroni and Valli (1994); Johnson (1987); Quarteroni et al (2007) for more information about the weak formulation and the numerical methods used to solve this kind of problem. For these equations, we use Darcy’s law module of COMSOL with a quadratic discretization. We solve these equations together using the fully coupled MUMPS direct solver.

### Numerical simulations—spherical geometry

**Fig. 2:**
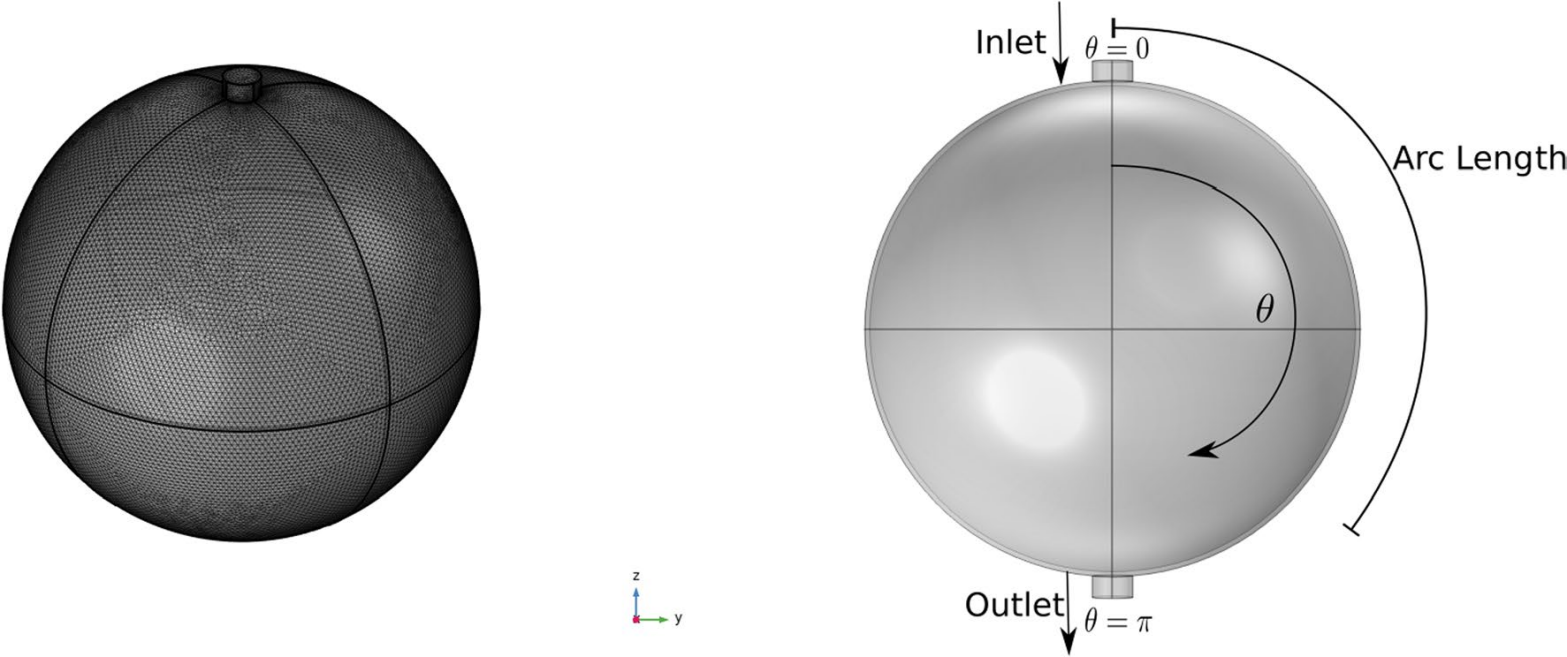
On the left, the mesh of the 3D simplified spherical geometry of our problem, inspired by a mouse popliteal lymph node as in Giantesio et al (2022); Girelli et al (2023). On the right, a representative plot of the geometric section parameters utilized throughout the entire paper

In this section, we numerically solve the model described in the previous sections in a simplified spherical geometry. We can see the 3D geometry of our problem in Fig. 2, where we refer to 2D concepts such as the polar angle and arc length because the 3D geometry exhibits symmetrical properties that allow for these 2D measurements to be relevant. Due to this symmetry, we have that the velocity is near zero at the axis of symmetry, i.e. at polar angle 0 and $\pi$ in accordance with the results found with the stream function approach used to solve the Stokes equation (see Giantesio et al 2022). The numerical results have been compared and validated with the analytical solution given in Girelli et al (2023).

**Fig. 3:**
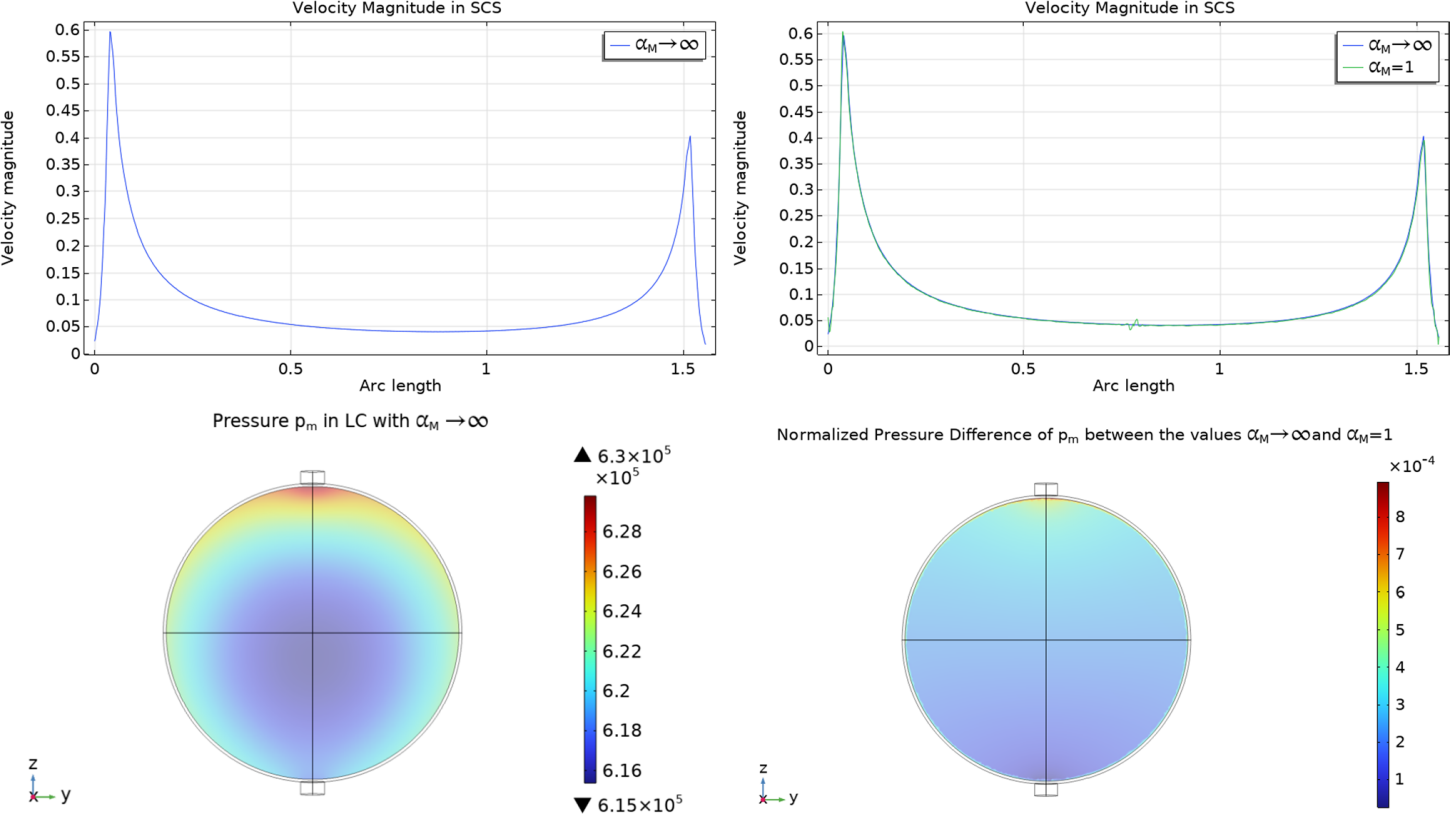
The first two plots (upper plots) represent the velocity magnitude in the SCS in $\frac{{{\text{mm}}}}{{\text{s}}}$ with respect to the arc length spanning the polar angle from 0 to $\pi$ as shown in Fig. 2, with two different value of $\alpha _M=1$ and $\alpha _M \rightarrow \infty$. The lower two plots represent the pressure values in the LC in mPa with $\alpha _M \rightarrow \infty$ and the pressure difference between the pressures $p_m$ with the values of $\alpha _M \rightarrow \infty$ and $\alpha _M=1$, normalized with respect to $p_\text {out}$. As we can see, the general value does not change much with respect to $\alpha _M$. Here we used the parameters $v_\text {in}=0.22\,\frac{{{\text{mm}}}}{{\text{s}}}$, $p_\text {out}=6.18\times 10^5$ mPa, $\pi _v-\pi _m= 1.02 \times 10^6$ mPa, $L_p=5.475 \times 10^{-11}\,\frac{\text {mm}}{\text {s mPa}}$ and ${\bar{p}}_v=1.06\times 10^6$ mPa

First of all, we want to see the effect of the Beavers-Joseph-Saffman parameter $\alpha _M$ in the interface condition (11). As we can see from the interface condition (11), we obtain the simplified interface condition (12) when $\alpha _M \rightarrow \infty$. In Fig. 3 we can see the velocity magnitude and the pressure values with $\alpha _M=1$ and $\alpha _M\rightarrow \infty$, and, as we can see, we have that this parameter does not influence much the velocity and the pressure in the whole domain (Shipley and Chapman 2010; Irons et al 2017). For this reason and for the reasons explained in Remark 1, from now on we fix the value $\alpha _M\rightarrow \infty$, which means we use the simplified interface condition (12).

**Fig. 4:**
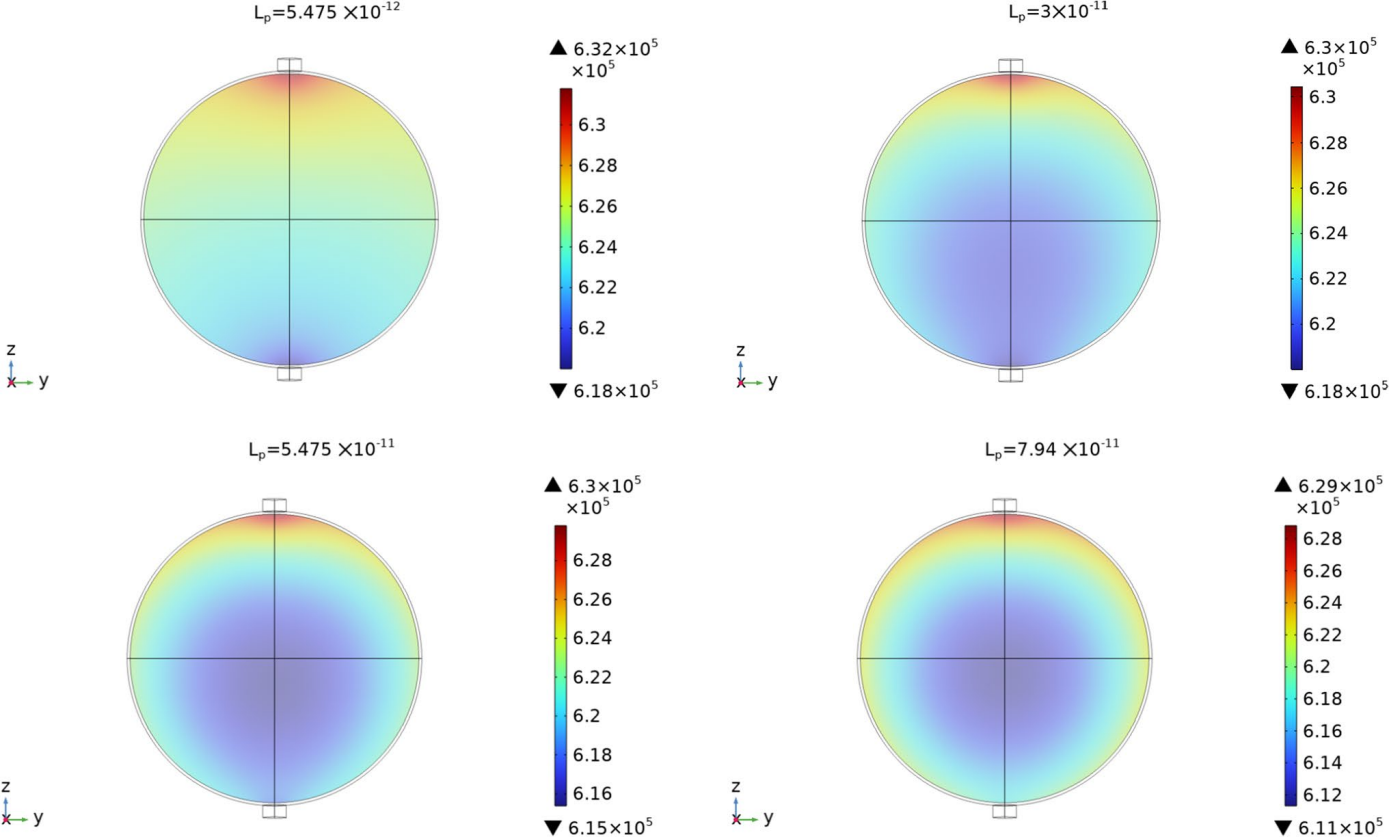
The interstitial pressure values $p_m$ in the LC with different values of $L_p$. Here we used the parameters $v_\text {in}=0.22\,\frac{{{\text{mm}}}}{{\text{s}}}$, $p_\text {out}=6.18\times 10^5$ mPa, $\pi _v-\pi _m= 1.02 \times 10^6$ mPa, and ${\bar{p}}_v=1.06\times 10^6$ mPa

In Fig. 4 we can see the interstitial pressure $p_m$ values in the LC varying the parameter $L_p$. We have similar behavior to the one found in Girelli et al (2023): increasing $L_p$ decreases the minimum of the interstitial pressure $p_m$ (and increasing the maximum of the blood vessels pressure $p_v$) and moves the minimum towards the center of the node. This behavior is due to a combination of the pressure variation given by the pressure of the Stokes flow in the SCS and the fluid exchange between phases. The values we found with these simulations are similar to the ones found in Girelli et al (2023) but slightly different: this is why it is important to take into account the coupling between the SCS and the LC.

**Fig. 5:**
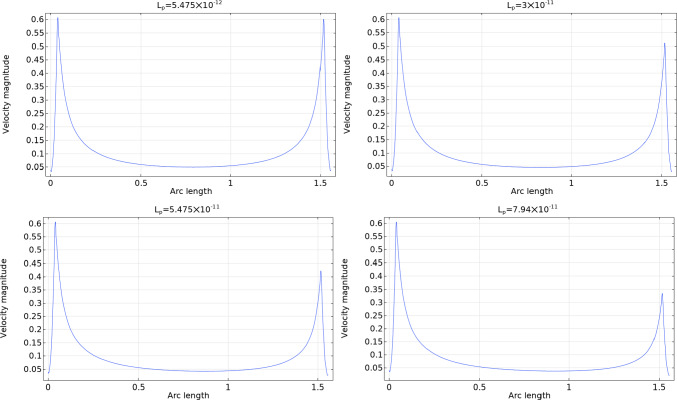
The velocity magnitude in the center of the SCS with respect to the arc length spanning the polar angle from 0 to $\pi$ as shown in Fig. 2, with different values of $L_p$. Here we used the parameters $v_\text {in}=0.22\,\frac{{{\text{mm}}}}{{\text{s}}}$, $p_\text {out}=6.18\times 10^5$ mPa, $\pi _v-\pi _m= 1.02 \times 10^6$ mPa, and ${\bar{p}}_v=1.06\times 10^6$ mPa

Given a uniform inlet velocity of $v_\text {in}=0.22\,\frac{{{\text{mm}}}}{{\text{s}}}$, the inlet fluid flow computed numerically is $\approx 1.083 \times 10^{-3}\,\frac{\text {mm}^3}{s}$ (with a relative error of about $1.5 \%$ from the value computed analytically of $1.1\times 10^{-3}\,\frac{\text {mm}^3}{s}$). Part of the lymph goes from the SCS to the LC (and then back to the blood circulation), and the remaining part goes out from the efferent lymphatic vessels: these quantities change with the parameter $L_p$, and we can see some results in Table 2. The sum of the columns “Outlet Flow” and “SCS $\rightarrow$ LC” must result approximately in the inlet fluid flow value $\approx 1.083 \times 10^{-3}\,\frac{\text {mm}^3}{s}$. As expected, increasing $L_p$ means increasing the fluid flow from the SCS to the LC, and it follows a lesser outlet fluid flow. We can see this behavior in Fig. 5, where the velocity near the efferent lymphatic vessel decreases as $L_p$ increases.

**Table 2:** Outlet fluid flow and the fluid flow passing through the external surface of the LC from the SCS in $\frac{\text {mm}^3}{s}$ varying the capillaries permeability $L_p$. Here we used the parameters $v_\text {in}=0.22\,\frac{{{\text{mm}}}}{{\text{s}}}$, $p_\text {out}=6.18\times 10^5$ mPa – $4\times 10^5$ mPa, $\pi _v-\pi _m= 1.02 \times 10^6$ mPa, and ${\bar{p}}_v=1.06\times 10^6$ mPa

$L_p$	Outlet Flow	SCS $\rightarrow$ LC	$p_\text {out}$
$5.475\times 10^{-12}\,\frac{\text {mm}}{\text {s mPa}}$	$1.05\times 10^{-3}\,\frac{\text {mm}^3}{s}$	$3.44\times 10^{-5}\,\frac{\text {mm}^3}{s}$	$6.18\times 10^5$ mPa
$1\times 10^{-11}\,\frac{\text {mm}}{\text {s mPa}}$	$1.02\times 10^{-3}\,\frac{\text {mm}^3}{s}$	$6.28\times 10^{-5}\,\frac{\text {mm}^3}{s}$	
$1.6\times 10^{-11}\,\frac{\text {mm}}{\text {s mPa}}$	$9.83\times 10^{-4}\,\frac{\text {mm}^3}{s}$	$1\times 10^{-4}\,\frac{\text {mm}^3}{s}$	
$3\times 10^{-11}\,\frac{\text {mm}}{\text {s mPa}}$	$8.97\times 10^{-4}\,\frac{\text {mm}^3}{s}$	$1.87\times 10^{-4}\,\frac{\text {mm}^3}{s}$	
$5.475\times 10^{-11}\,\frac{\text {mm}}{\text {s mPa}}$	$7.44\times 10^{-4}\,\frac{\text {mm}^3}{s}$	$3.4\times 10^{-4}\,\frac{\text {mm}^3}{s}$	
$7.94\times 10^{-11}\,\frac{\text {mm}}{\text {s mPa}}$	$5.93\times 10^{-4}\,\frac{\text {mm}^3}{s}$	$4.9\times 10^{-4}\,\frac{\text {mm}^3}{s}$	
$5.475\times 10^{-12}\,\frac{\text {mm}}{\text {s mPa}}$	$1.065\times 10^{-3}\,\frac{\text {mm}^3}{s}$	$1.84\times 10^{-5}\,\frac{\text {mm}^3}{s}$	$4\times 10^5$ mPa
$1\times 10^{-11}\,\frac{\text {mm}}{\text {s mPa}}$	$1.058\times 10^{-3}\,\frac{\text {mm}^3}{s}$	$3.35\times 10^{-5}\,\frac{\text {mm}^3}{s}$	
$1.6\times 10^{-11}\,\frac{\text {mm}}{\text {s mPa}}$	$1.01\times 10^{-3}\,\frac{\text {mm}^3}{s}$	$5.35\times 10^{-5}\,\frac{\text {mm}^3}{s}$	
$3\times 10^{-11}\,\frac{\text {mm}}{\text {s mPa}}$	$9.83\times 10^{-4}\,\frac{\text {mm}^3}{s}$	$1\times 10^{-4}\,\frac{\text {mm}^3}{s}$	
$5.475\times 10^{-11}\,\frac{\text {mm}}{\text {s mPa}}$	$8.8\times 10^{-4}\,\frac{\text {mm}^3}{s}$	$1.82\times 10^{-4}\,\frac{\text {mm}^3}{s}$	
$7.94\times 10^{-11}\,\frac{\text {mm}}{\text {s mPa}}$	$8\times 10^{-4}\,\frac{\text {mm}^3}{s}$	$2.62\times 10^{-4}\,\frac{\text {mm}^3}{s}$	

We observe that varying the other parameters gives results with behavior similar to what we found in Girelli et al (2023).

**Fig. 6:**
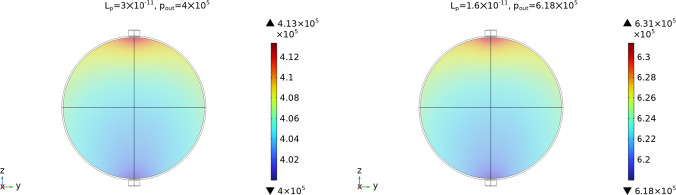
The pressure values in the LC with different values of $p_\text {out}$ and $L_p$ but with the same fluid flow values of Tables 2 and 3.1. Here we used the parameters $v_\text {in}=0.22\,\frac{{{\text{mm}}}}{{\text{s}}}$, $\pi _v-\pi _m= 1.02 \times 10^6$ mPa, and ${\bar{p}}_v=1.06\times 10^6$ mPa

The plots and the data above are obtained with $p_\text {out}=6.18\times 10^5$ mPa, that is a value inspired by the experiments of Bouta et al (2014) (the minimum of the value range); in Jafarnejad et al (2015) they used a value of $p_\text {out}=4\times 10^5$ mPa, and in Cooper et al (2016, 2018) they used a value of $p_\text {out}=0$ mPa. Considering that the value of the pressure is important to study the fluid exchange between phases, we want to see the differences between using a different outlet pressure. Hence now we fix $p_\text {out}=4\times 10^5$ mPa. In the second part of Table 2 we can see the fluid flow computed with different $L_p$ in this case; as we can see, to have the same outlet fluid flow (and the same SCS $\rightarrow$ LC fluid flow) as in the case with $p_\text {out}=6.18\times 10^5$ mPa, we need a higher value of $L_p$. If we fix the same outlet fluid flow value of $\approx 9.83 \times 10^{-4}\,\frac{\text {mm}^3}{s}$ (that is chosen by the fact that more than $90 \%$ of the lymph remain in the SCS without entering the LC (Jafarnejad et al 2015)), we have $L_p=1.6\times 10^{-11}\,\frac{\text {mm}}{\text {s mPa}}$ for $p_\text {out}=6.18\times 10^5$ mPa and a value of $L_p=3\times 10^{-11}\,\frac{\text {mm}}{\text {s mPa}}$ for $p_\text {out}=4\times 10^5$ mPa. As we can see in Fig. 6, with these different values we have the same pressure behavior and range but with different pressure values.

**Fig. 7:**
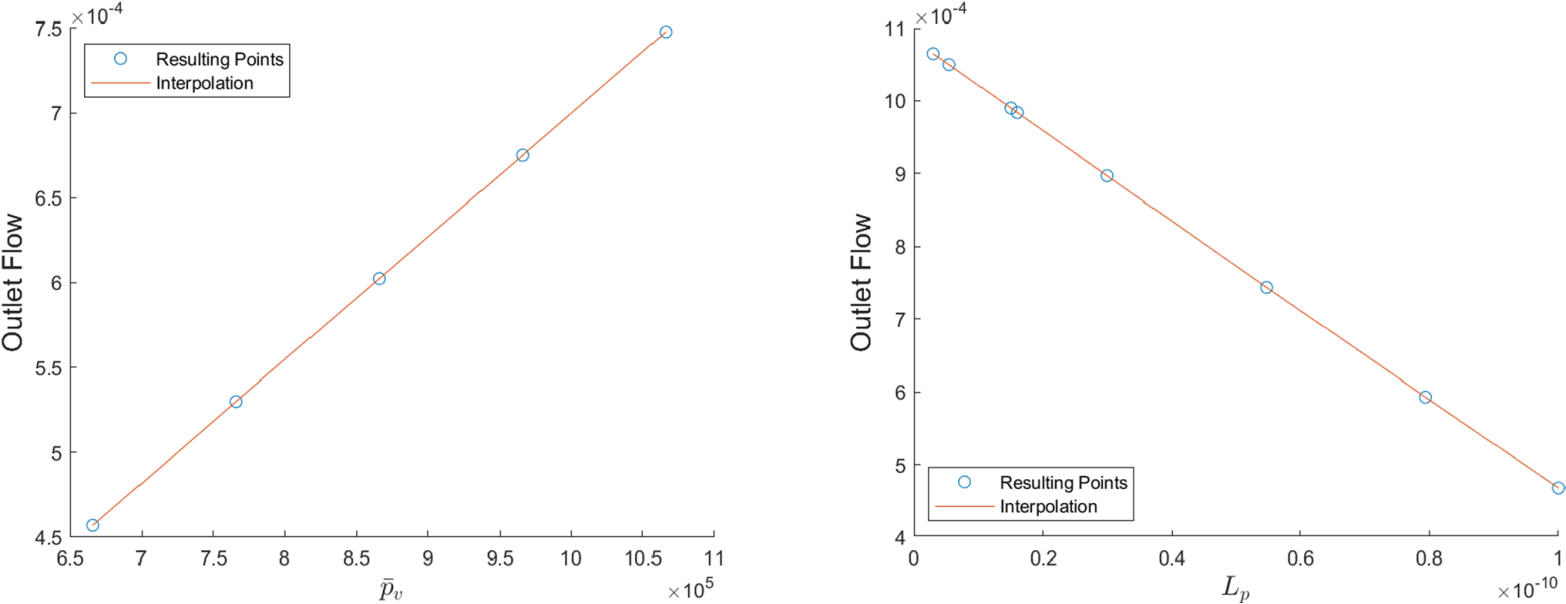
The outlet flow computed in the efferent lymphatic vessel with different values of ${\bar{p}}_v$ (on the left) and $L_p$ (on the right), with the fluid flow values of Table 1. Here we used the parameters $v_\text {in}=0.22\,\frac{{{\text{mm}}}}{{\text{s}}}$, $\pi _v-\pi _m= 1.02 \times 10^6$ mPa, $L_p=5.475\times 10^{-11}\,\frac{\text {mm}}{\text {s mPa}}$ (for the plot on the left) and ${\bar{p}}_v=1.06\times 10^6$ mPa (for the plot on the right)

In Fig. 7 we can see the outlet flow computed in the efferent lymphatic vessel varying ${\bar{p}}_v$ and $L_p$. As we can see, increasing $L_p$ results in decreasing the outlet flow (and a consequent increase of the fluid that goes into the LC, as we can see above); instead, increasing ${\bar{p}}_v$ results in increasing the outlet flow (and a consequent decrease of the fluid that goes into the LC). Moreover, we can see that there is a linear relation between the outlet flow computed and the variation of the parameters ${\bar{p}}_v$ and $L_p$: a similar behavior is reported in Jafarnejad et al (2015).

From the simulations we can estimate the pressure ${\bar{p}}_v$ for which we have an inversion of the fluid exchange flow direction: when we have $p_\text {out}=6.18\times 10^5\,$ mPa, we have a flow inversion at ${\bar{p}}_v=1.54\times 10^6\,\text {mPa}\approx 11.6$ mmHg, similar to the ones found in Girelli et al (2023); instead, for $p_\text {out}=4\times 10^5\,$ mPa, we have a flow inversion at ${\bar{p}}_v=1.35\times 10^6\,\text {mPa}\approx 10.1$ mmHg, similar to the one found in Jafarnejad et al (2015).

**Fig. 8:**
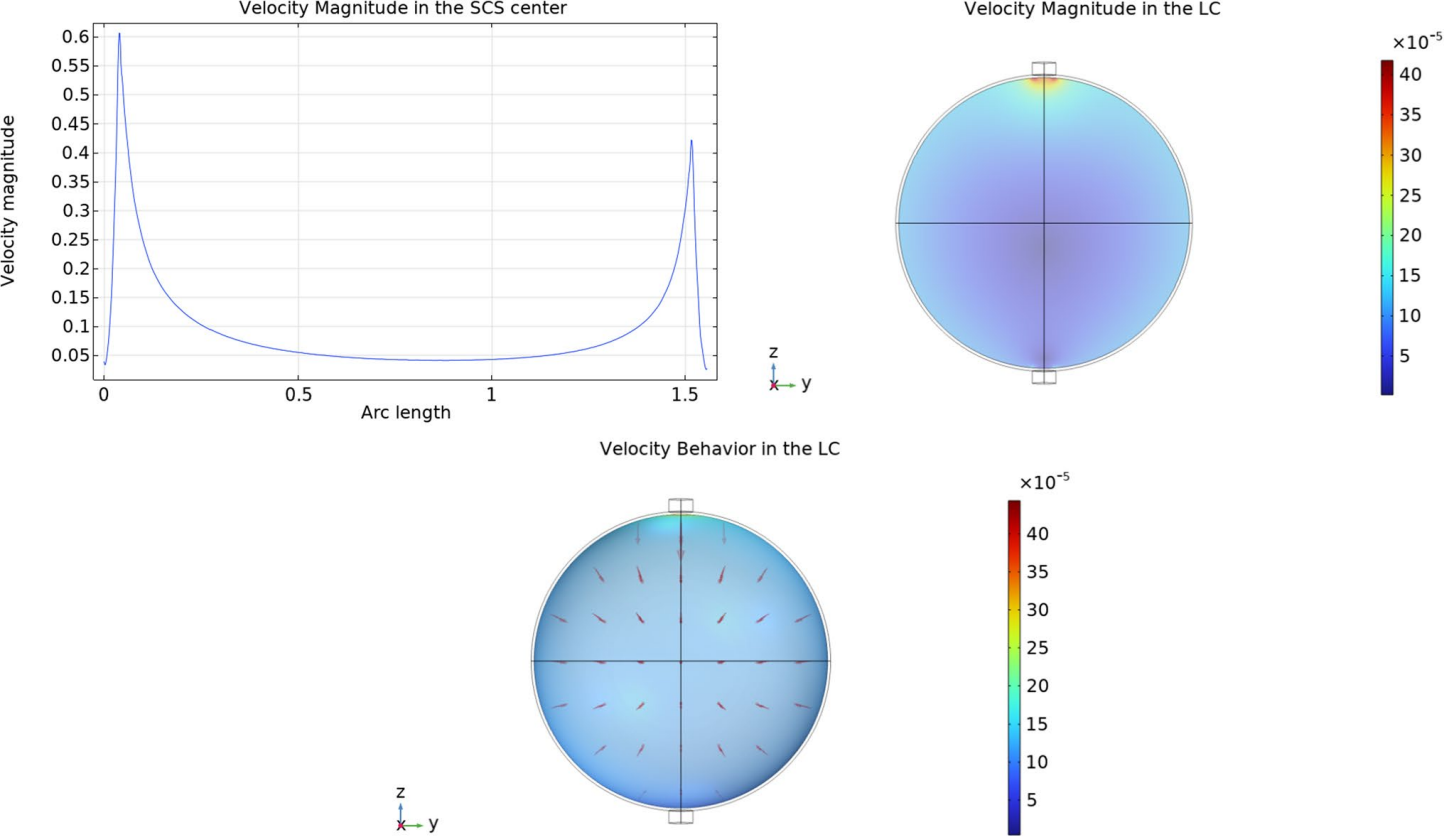
The velocity magnitude computed in the subcapsular sinus center with respect to the spherical arc length spanning the polar angle from 0 to $\pi$ as shown in Fig. 2 (upper on the left) and on the lymphoid compartment (upper on the right) and the velocity magnitude together with the velocity arrows in the LC (lower), with the fluid flow values of Tables 2. Here we used the parameters $v_\text {in}=0.22\,\frac{{{\text{mm}}}}{{\text{s}}}$, $\pi _v-\pi _m= 1.02 \times 10^6$ mPa, $L_p=5.475\times 10^{-11}\,\frac{\text {mm}}{\text {s mPa}}$ and ${\bar{p}}_v=1.06\times 10^6$ mPa

In Fig. 8 we can see the velocity magnitude and the velocity behavior inside the lymph node: as we can see, the velocity inside the porous bulk region (the lymphoid compartment) is extremely lower with respect to the one in the subcapsular sinus. The velocities that we found in our simulations inside the lymphoid compartment are in agreement with the founding in the literature, where the velocity range from $1.5\times 10^{-5}\,\frac{{{\text{mm}}}}{{\text{s}}}$ to $6\times 10^{-4}\,\frac{{{\text{mm}}}}{{\text{s}}}$ (Shanti et al 2020; Chary and Jain 1989; Jafarnejad et al 2015; Tomei et al 2009; Dafni et al 2002). If we compare this solution with the one that we found analytically in Girelli et al (2023) using a given pressure distribution found by the stream function approach in Giantesio et al (2022), the qualitative behavior remains the same (a higher pressure near the inlet, a lower pressure near the outlet, and a lower pressure region near the center of the node, and the same for the velocity), but here we have a higher maximum velocity respect to the values we found analytically: this is because here we couple the fluid flow in the SCS with the fluid flow in the LC, and this allows us to find more precise boundary data for the LC.

### Numerical simulations—oblate spheroidal geometry

In this section we numerically solve the model presented in the previous sections in a more realistic lymph node geometry. Indeed, a lymph node generally has an oblate spheroidal shape (Birmingham et al 2020; Jafarnejad et al 2015; Tretiakova et al 2021; Giantesio et al 2021). We represent this geometry in the $x-z$ plane in this way

15 $$\begin{aligned} \begin{bmatrix} x \\ z \end{bmatrix}=\begin{bmatrix} a \cos \theta \\ b \sin \theta \end{bmatrix}, \end{aligned}$$

with major semiaxis $a=0.5$ mm, minor semiaxis $b=0.35$ mm (Jafarnejad et al 2015), and subcapsular sinus thickness $h=10$ $\mu$m (Jafarnejad et al 2015; Giantesio et al 2022). It follows that the parametric equation that describes the LC geometry on a $x-z$ plane is

16 $$\begin{aligned} \begin{bmatrix} x \\ z \end{bmatrix}=\begin{bmatrix} a \cos \theta \\ b \sin \theta \end{bmatrix}-h\frac{\begin{bmatrix} \frac{1}{a^2}\cos \theta \\ \frac{1}{b^2}\sin \theta \end{bmatrix}}{\sqrt{ \frac{\cos ^2\theta }{a^4}+ \frac{\sin ^2\theta }{b^4}}}. \end{aligned}$$

**Fig. 9:**
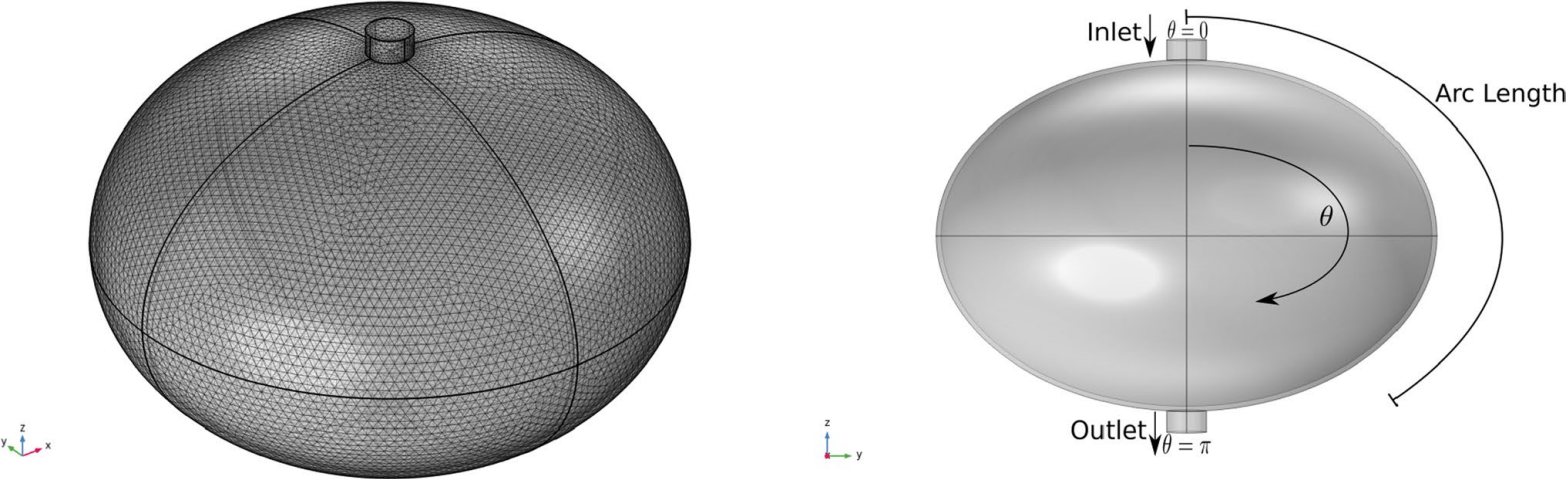
On the left, the mesh of the 3D geometry of our problem, inspired by a mouse popliteal lymph node (Jafarnejad et al 2015). On the right, a representative plot of the geometric section parameters utilized throughout the entire paper

We can see the 3D geometry in Fig. 9 (where we rotate the 2D geometry described above with respect to the *z*-axis).

As in Sect. 3.1, we use the no-slip interface condition (12) because, as happens in the spherical case, the behavior of the result with different $\alpha _M$ is very similar.

**Fig. 10:**
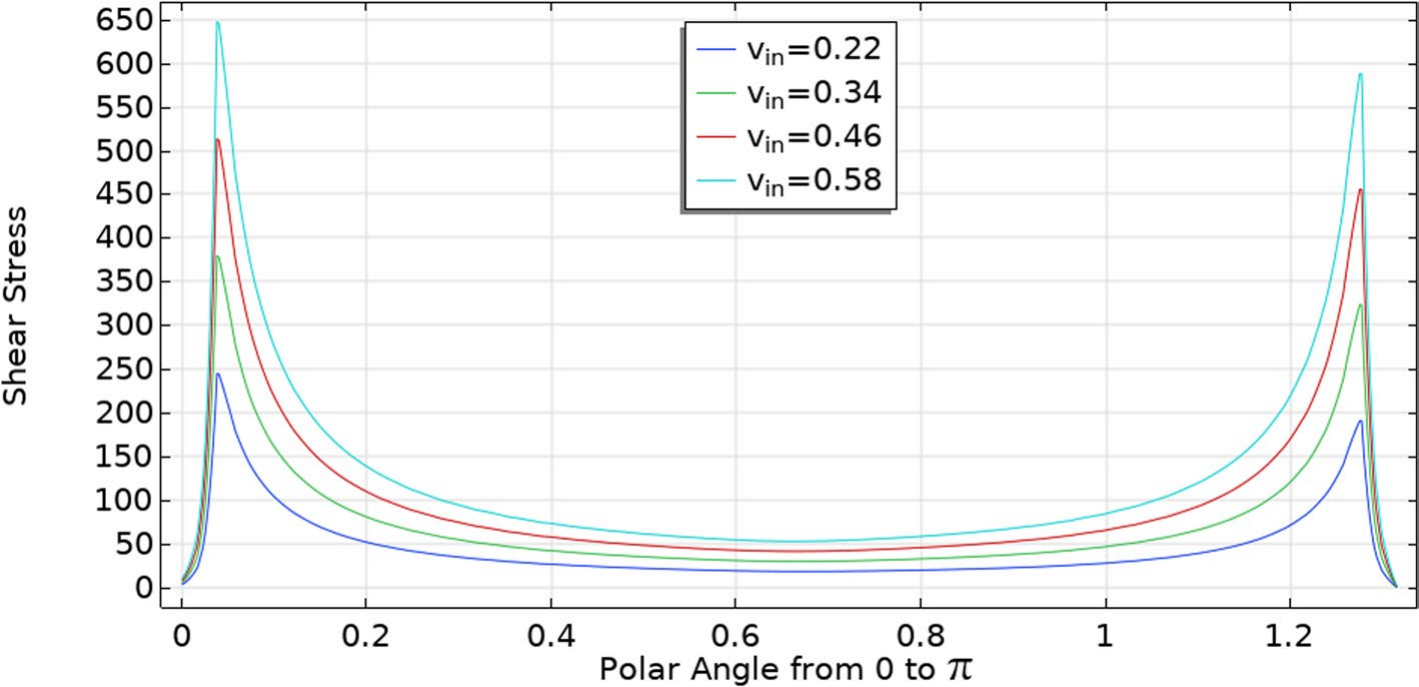
Shear stress at the interface between the SCS and the LC in mPa varying the inlet velocity (in $\frac{{{\text{mm}}}}{{\text{s}}}$) with respect to the spheroidal arc length spanning the ellipsoidal angle from 0 to $\pi$ as shown in Fig. 9. We use these chosen parameters: $p_\text {out}=6.18\times 10^5$ mPa, $\pi _v-\pi _m= 1.02 \times 10^6$ mPa, $L_p=5.475 \times 10^{-11}\,\frac{\text {mm}}{\text {s mPa}}$ and ${\bar{p}}_v=1.06\times 10^6$ mPa

We can see the shear stress at the interface between the SCS and the LC in Fig. 10 with respect to the arc length of the interface varying the inlet velocity. As we can see, we have the maximum shear stress near the inlet (arc length near zero) and then, near the outlet (arc length near $\pi$) we have a local maximum but smaller than the inlet one; this happens because part of the lymph “vanishes” from the lymph node due to the fluid exchange with the blood vessels inside it and this result in a lesser outlet flow (and lesser shear stress near the outlet). Increasing $v_\text {in}$ also increases shear stress; for the shear stress curve obtained with $v_\text {in}=0.58\,\frac{{{\text{mm}}}}{{\text{s}}}$, we obtain the same behavior and values obtained in Jafarnejad et al (2015). The importance of this behavior at the interface lies in its direct connection to cell adhesion on the exterior of the LC, which correlates directly with shear stress (Birmingham et al 2020). Furthermore, it is worth noting that shear stress also plays a crucial role in certain pathologies, for instance, B-cell lymphoma (Apoorva et al 2018).

**Fig. 11:**
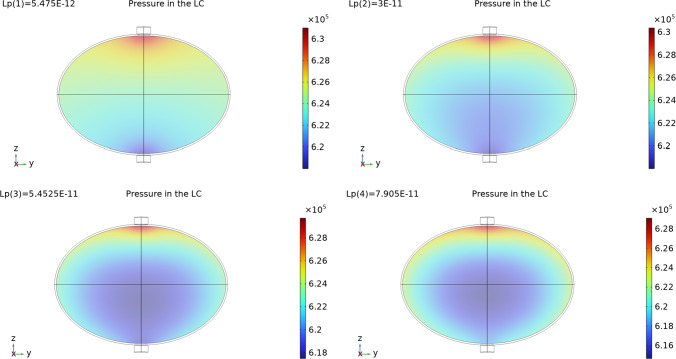
The interstitial pressure values $p_m$ in the LC with different values of $L_p$. Here we used the parameters $v_\text {in}=0.22\,\frac{{{\text{mm}}}}{{\text{s}}}$, $p_\text {out}=6.18\times 10^5$ mPa, $\pi _v-\pi _m= 1.02 \times 10^6$ mPa, and ${\bar{p}}_v=1.06\times 10^6$ mPa

**Fig. 12:**
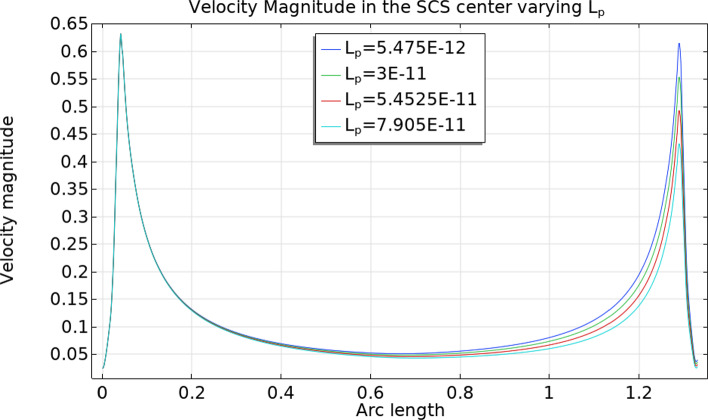
The velocity magnitude (in mm/s) in the SCS center with respect to the spheroidal arc length spanning the ellipsoidal angle from 0 to $\pi$ (as shown in Fig. 9) with different values of $L_p$. Here we used the parameters $v_\text {in}=0.22\,\frac{{{\text{mm}}}}{{\text{s}}}$, $p_\text {out}=6.18\times 10^5$ mPa, $\pi _v-\pi _m= 1.02 \times 10^6$ mPa, and ${\bar{p}}_v=1.06\times 10^6$ mPa

In Fig. 11 we can see the interstitial pressure behavior with different $L_p$. As we can see, increasing $L_p$ results in a decrease of the minimum pressure and the moving of this minimum towards the center of the node. This behavior means that, as $L_p$ increases, more lymph moves from the lymph node to the blood vessels inside it, resulting in a lesser outlet fluid flow. We can see this behavior in Fig. 12. This is the same behavior that we found in the spherical case. Varying the other parameters results in the similar behavior we found for the spherical case in Sect. 3.1 and in Girelli et al (2023). Moreover, we have that with the same value of $L_p$, for the spheroidal case we have a higher outlet fluid flow. In particular, if we fix, for instance, the value $L_p=3 \times 10^{-11}\,\frac{\text {mm}}{\text {s mPa}}$, we have for the spherical case an outlet flow of $8.97\times 10^{-4}\,\frac{\text {mm}^3}{\text {s}}$ (see Table 2), instead for the spheroidal case we have an outlet flow of $9.6\times 10^{-4}\,\frac{\text {mm}^3}{\text {s}}$.

**Fig. 13:**
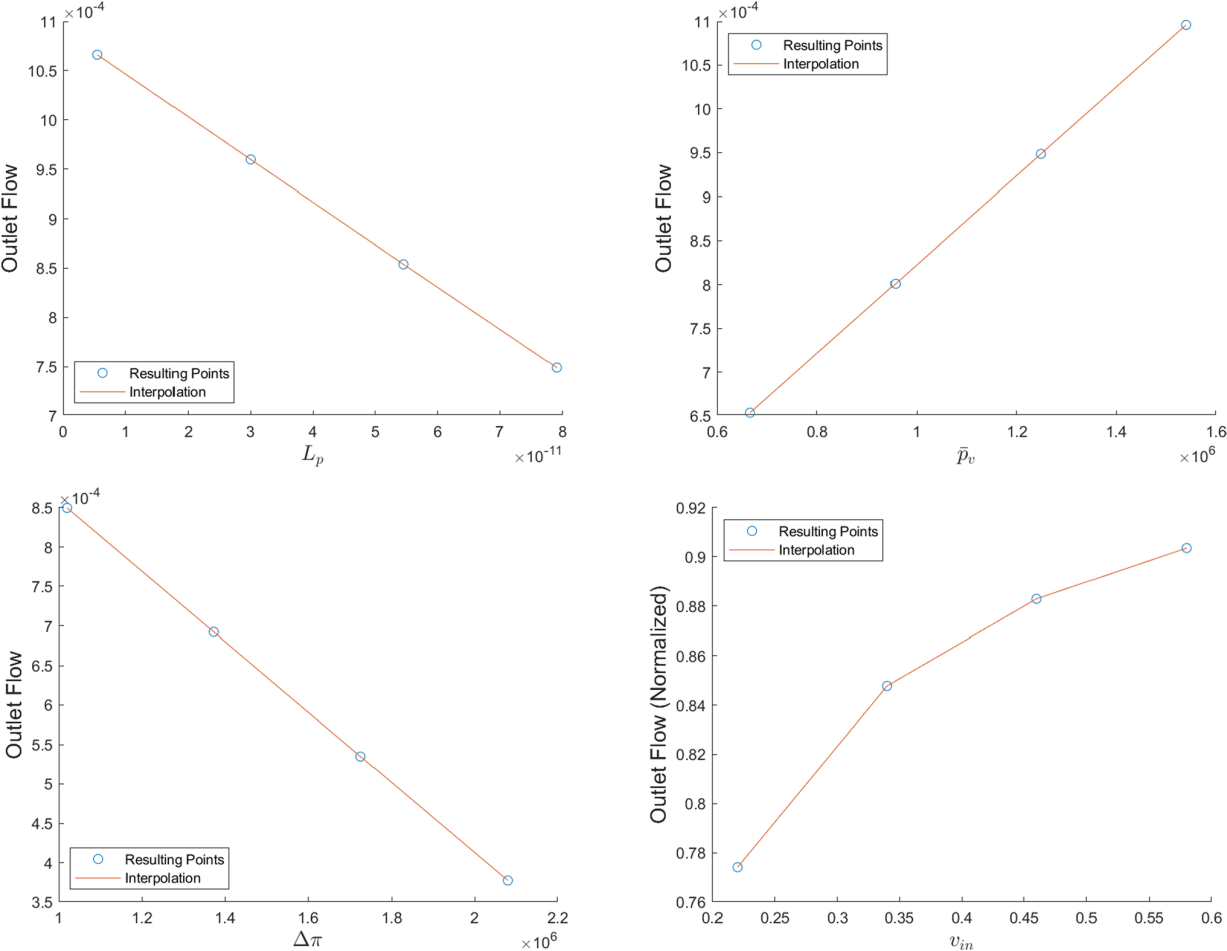
The outlet flow (in $\frac{\text {mm}^3}{s}$) computed in the efferent lymphatic vessel with different values of $L_p$ (upper-left), ${\bar{p}}_v$ (upper right), $\Delta \pi$ (lower-left), and $v_\text {in}$ (lower-right, here the outlet flow is normalized with respect to the flow values of $\pi R_{LV}^2v_\text {in}$, and it is dimensionless) with the fluid flow values of Table 1. Here we used the parameters (when not varying) $v_\text {in}=0.22\,\frac{{{\text{mm}}}}{{\text{s}}}$, $\pi _v-\pi _m= 1.02 \times 10^6$ mPa, $L_p=5.475\times 10^{-11}\,\frac{\text {mm}}{\text {s mPa}}$ and ${\bar{p}}_v=1.06\times 10^6$ mPa

We can see better how the parameters that regulate the fluid exchange between the lymph and the blood vessels affect the outlet fluid flow in the plots of Fig. 13. As we can see, increasing $L_p$ and $\Delta \pi$ results in a linear decrease of the outlet fluid flow, meaning that more lymph moves in the blood vessels; instead, increasing $\bar{p}_v$ results in a linear increase of the outlet fluid flow, meaning that less lymph moves inside the blood vessels. This behavior is in agreement with the findings of Sect. 3.1 and of Girelli et al (2023); Jafarnejad et al (2015). Moreover, for $p_\text {out}=6.18\times 10^5\,$ mPa, we have a flow inversion at ${\bar{p}}_v=1.54\times 10^6\,\text {mPa}\approx 11.6\,\text {mmHg}$, similar to the ones found in Girelli et al (2023) and the same we found for the spherical case.

The last plot (lower-right) of Fig. 13 describes the variation of the outlet flow with respect to the inlet velocity $v_\text {in}$. In this case, we normalize the outlet flow with respect to the inlet flow (computed as $\pi R_{LV}^2v_\text {in}$) to see the % of the fluid that reaches the efferent lymphatic vessel; moreover, it is obvious that increasing the inlet flow results in an increasing of the outlet flow too, therefore, normalization is performed to mitigate the presence of this behavior as well. As we can see, increasing the inlet velocity $v_\text {in}$ results in an increase of the normalized outlet flow, meaning that a greater % of the lymph reaches the efferent vessel. This happens because increasing the inlet velocity means that the residence time of the lymph in the node decreases, which means a lesser time for fluid exchange inside the node. This result is found experimentally in Adair et al (1982).

**Fig. 14:**
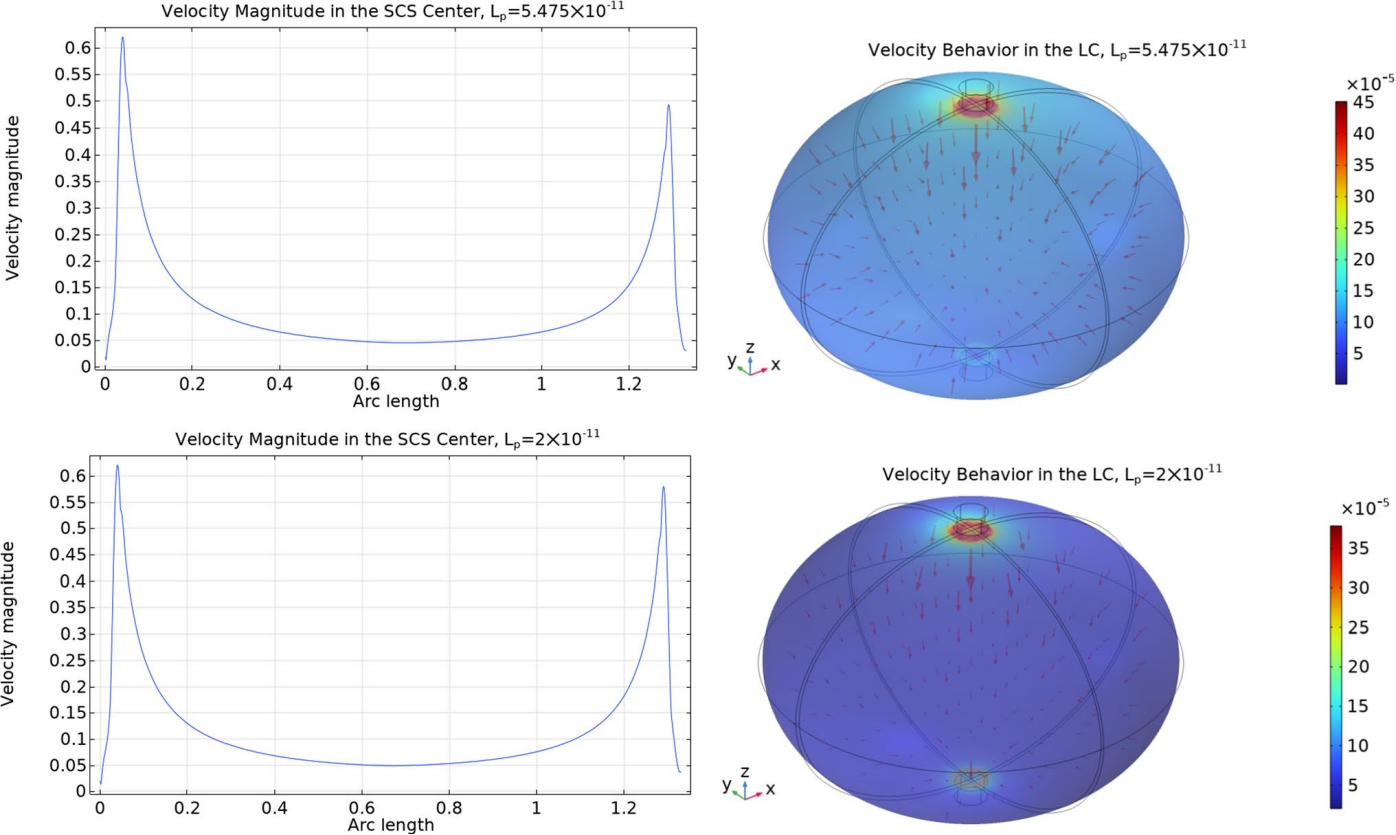
The velocity magnitude (in mm/s) computed at the center of the subcapsular sinus with respect to the spheroidal arc length spanning the ellipsoidal angle from 0 to $\pi$ as shown in Fig. 9 (left) and the velocity magnitude together with the velocity arrows in the LC (right), with the fluid flow values of Tables 2 in a spheroidal geometry, with two different values of $L_p$ (in $\frac{\text {mm}}{\text {s mPa}}$). Here we used the parameters $v_\text {in}=0.22\,\frac{{{\text{mm}}}}{{\text{s}}}$, $\pi _v-\pi _m= 1.02 \times 10^6$ mPa, and ${\bar{p}}_v=1.06\times 10^6$ mPa

In Fig. 14 we can see the velocity behavior inside both the SCS and the LC with two different values of $L_p$: $L_p=5.475\times 10^{-11}\,\frac{\text {mm}}{\text {s mPa}}$ is the same value that we used in Fig. 8 of Sect. 3.1, and $L_p=2\times 10^{-11}\,\frac{\text {mm}}{\text {s mPa}}$ is the value for which about 90% of the afferent lymph goes out of the lymph node from (as found in Jafarnejad et al 2015). As in the spherical case, the velocity inside the lymphoid compartment is extremely lower with respect to the one in the subcapsular sinus. The biological motivation is that B cells seem to engage in a progressive buildup of antigens over time, rather than experiencing instant activation upon encountering antigens. This implies the occurrence of multiple cycles of antigen acquisition, as indicated by Carrasco and Facundo (2007). The significance of the porous region’s remarkably low velocity becomes evident, as it grants ample time for both antigens and cells carrying antigens to locate lymphocytes and initiate their activation, a point emphasized by Shanti et al (2020). Moreover, the maximum velocity inside the LC (in the region near the inlet condition) in the spheroidal case is slightly bigger than the spherical one but remains in the literature range from $1.5\times 10^{-5}\,\frac{{{\text{mm}}}}{{\text{s}}}$ to $6\times 10^{-4}\,\frac{{{\text{mm}}}}{{\text{s}}}$ (Shanti et al 2020; Chary and Jain 1989; Jafarnejad et al 2015; Tomei et al 2009; Dafni et al 2002). Between the two plots with different $L_p$, the maximum velocity of the case with a smaller $L_p$ is lesser than the one with a higher $L_p$: this is consistent because less fluid enters the LC when $L_p$ is smaller.

## Cell problem numerical simulations

**Fig. 15:**
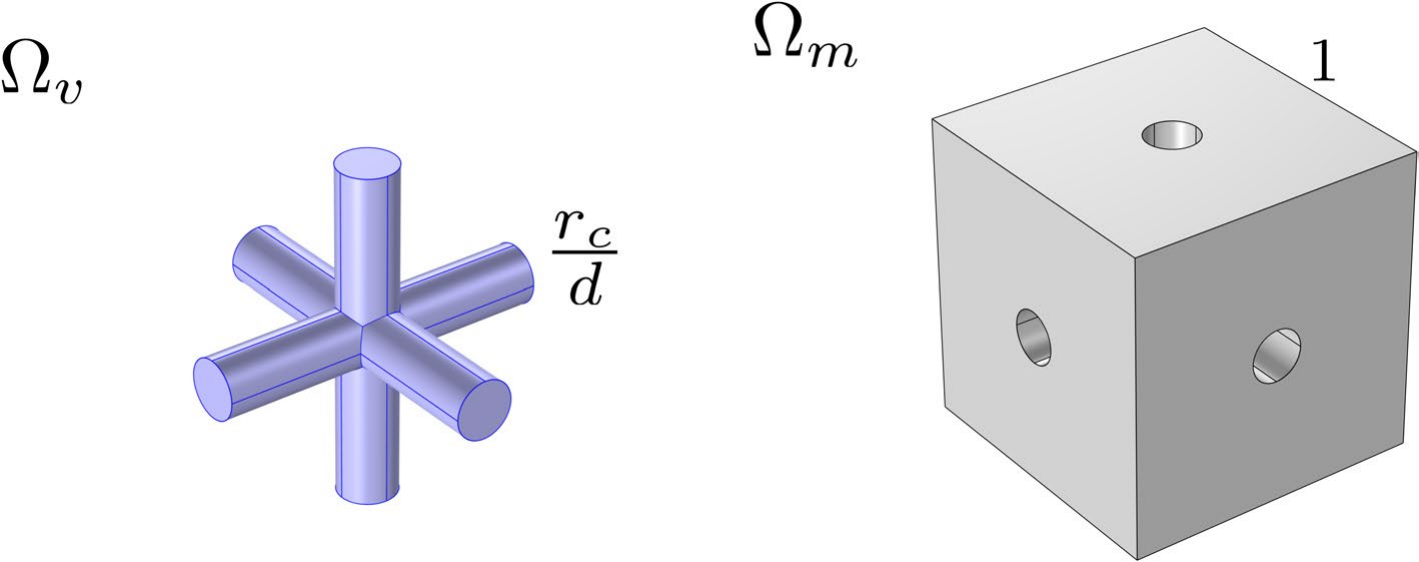
The cell problem domains $\Omega _v$ (left) and $\Omega _m$ (right) in a non-dimensional form. The normalized cube has side 1 and the normalized tricylinder has radius ${\bar{r}}=r_c/d$

In this section, we recall and discuss the numerical simulations used to solve the cell problems (5) and (8) in the geometry represented in Fig. 15 and with the data of Table 1.

We assume that both porous media are isotropic, hence solutions of (5) and (8) becomes

$$\begin{aligned} \varvec{W}_m=W_m{\mathbb {I}}, \quad \nabla _{\varvec{x}}g_v=G_v{\mathbb {I}}, \end{aligned}$$

where $W_m$ and $G_v$ are constants due to the hypotheses used.

**Fig. 16:**
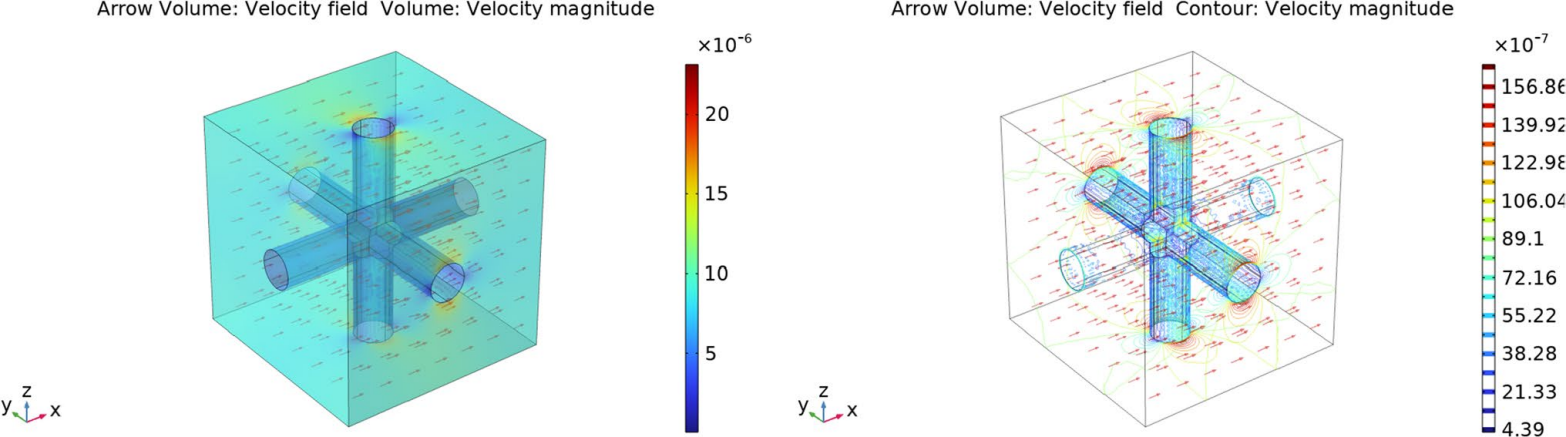
The velocity solution of cell problem (5) in the geometry $\Omega _m$ in a non-dimensional form using the physiological data of Table 1

We solve these cell problems using COMSOL Multiphysics in the same way as we did in Girelli et al (2023, Appendix C). We report the methods and the results here for the readers’ convenience. To address the cell problem described by equation (5) within the geometry $\Omega _m$, we employ the COMSOL Brinkman equations module, using a PARDISO solver. Moreover, we use a ${\mathbb {P}}^3_2-{\mathbb {P}}_1$ discretization for the fluid and pressure variables, respectively. Figure 16 displays the velocity solution in the $\varvec{e}_1$ direction. It is noteworthy that the solution remains the same across all directions due to the symmetry of the geometry and the isotropy of the porous medium.

The value of the hydraulic conductivity $\langle W_m\rangle _{\Omega _m}$ in (3) computed by our simulation is

17 $$\begin{aligned} \langle W_m\rangle _{\Omega _m}\approx 9.1163\times 10^{-6}. \end{aligned}$$

We perform an adaptive mesh refinement study to analyze the mesh used in our simulation, and we find a value of

18 $$\begin{aligned} \langle W^\text {ref}_m\rangle _{\Omega _m}\approx 9.1187\times 10^{-6}, \end{aligned}$$

giving a relative error of $\approx 0.026 \%$.

**Fig. 17:**
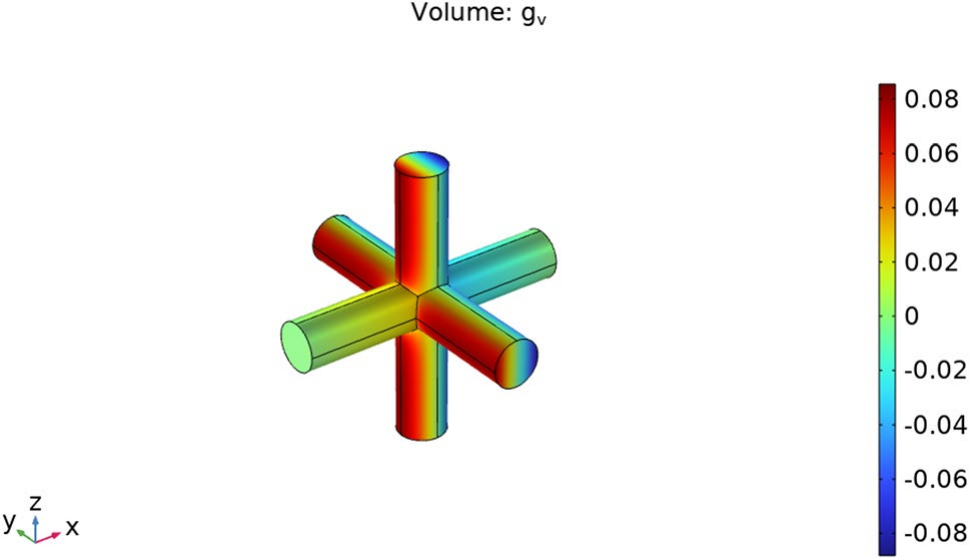
The solution of cell problem (8) in the geometry $\Omega _v$ in a non-dimensional form using the physiological data found in Table 1

The cell problem expressed by equation (8) within the geometry $\Omega _v$ takes the form of Poisson’s equation. To solve it, we employ COMSOL Poisson’s equation module using quadratic element order for discretization, and we use MUMPS as the solver. We can see the solution in Fig. 17 computed in the direction $\varvec{e}_1$ (as in the previous case, we have the same solution for every direction).

The value $\langle G_v\rangle _{\Omega _v}$ computed by our simulations for the hydraulic conductivity (6) is

19 $$\begin{aligned} \langle G_v\rangle _{\Omega _v}\approx -0.60060. \end{aligned}$$

Performing an adaptive mesh refinement study for this problem we find a value of

20 $$\begin{aligned} \langle G^\text {ref}_v\rangle _{\Omega _m}\approx -0.60054, \end{aligned}$$

giving a relative error of $\approx 0.01 \%$.

## Conclusions

In this paper, we have presented some numerical results that describe the fluid flow in an entire lymph node. The scope of the paper was to couple the subcapsular sinus (free fluid region) with the model for the lymphoid compartment (porous bulk region) (Girelli et al 2023) in a geometry more similar to a real lymph node to study in more detail the fluid flow inside the whole system. For this purpose, we have performed numerical simulations to study the behavior of the lymph inside the lymph node in different cases. In Sects. 2 and 3 we have studied the fluid flow using the Stokes equation in the subcapsular sinus (free-fluid region) and the model found in Girelli et al (2023) for the porous bulk region (lymphoid compartment). In particular, we have used two different geometries: the simplified spherical geometry to compare the results with the analytical founding in Girelli et al (2023), and an oblate spheroidal geometry, which is more realistic to describe the lymph node (Jafarnejad et al 2015; O’Melia et al 2019; Tretiakova et al 2021; Giantesio et al 2021; Birmingham et al 2020; Shanti et al 2020) and to see the impact in using different geometries.

We have compared the results found in this paper with both data and findings available in the lymph node literature, and we have found that our results are in line with these data. Thanks to these simulations, we can study the lymph inside the lymph node in a more general and realistic geometry, and this affects the fluid behavior inside the node.

From these simulations, we were able to confirm that, even though the pressure in the blood vessels is higher than the interstitial pressure within the node, lymph flows from the node into the bloodstream. This happens because the blood vessels have a higher protein concentration with respect to the lymph. In our model, this behavior is represented by a sink term in the LC of the node, as we have shown in different plots of the solution. This sink term contributes to the motion of lymph within the lymph node along with the pressure gradient generated by the movement of lymph within the SCS, thereby creating an intermediate situation between these two phenomena, regulated by the microscale interfacial properties between blood vessels and lymph. This clearly shows that the multiscale properties of the lymph node are highly significant. Furthermore, it seems that this phenomenon occurring within the lymph node has been crucial for the balance and regulation of fluid within the lymphatic system. Indeed, damage or removal of lymph nodes leads to a situation called lymphœdema, which is connected to an impairment of lymphatic transport (Moore Jr and Bertram 2018; Tobbia et al 2009). Finally, understanding the biophysical forces and the lymph movement inside the node can help in understanding the immune and drug transport in the whole lymphatic system (Arasa et al 2021; O’Melia et al 2019; Birmingham et al 2020; Shanti et al 2020). In particular, a non-functioning lymphatic system can lead to a severe increase in the interstitial pressure which can in turn impair blood and drug convection within biological systems affected by cancer diseases, see, e.g., Jain et al (2007).

The current work is open for improvements. First, we can take into account the time behavior of the lymph inside the node, so that we can impose a pulsatile inlet condition for the velocity to mimic the lymphangion contraction (Girelli et al 2024). Moreover, it could be interesting to couple the fluid flow motion in the lymphangion and the lymph node together.

A very interesting extension of this model would be to incorporate the temporal and spatial dependence of protein and drug concentrations within the node, in both the FRC and the blood vessels network, to allow a more detailed description of the fluid exchange between these two phases (Penta et al 2015).

We simplified the model presented in Girelli et al (2023) by assuming that multiscale forces were both zero; such forces can play a significant role, especially when utilizing electromagnetic fields (for example in cancer hyperthermia, see Penta 2022; Al Sariri et al 2023). Therefore, it is essential to consider the influence of inhomogeneous volume loads when we get access to physiological data, as outlined in Penta et al (2020).

To simplify the model and address the scarcity of relevant biological data, we employed a rigid porous matrix in this study. However, a possible improvement for this model in the future could involve integrating a deformable matrix that interacts with the lymph flow within the node.

Finally, we opted for an ellipsoidal shape (Jafarnejad et al 2015; Cooper et al 2016, 2018; Giantesio et al 2021; Tretiakova et al 2021; O’Melia et al 2019; Shanti et al 2020). Acquiring more precise data on the lymph node morphology, potentially through the use of medical imaging techniques, could facilitate the refinement of our modeling approach, enabling us to numerically compute macroscopic solutions. This advancement would empower us to generate meaningful physiological predictions in the future.

## Data Availability

Not applicable.
